# Coelenterazine Indicators for the Specific Imaging of Human and Bovine Serum Albumins

**DOI:** 10.3390/s23136020

**Published:** 2023-06-29

**Authors:** Sung-Bae Kim, Genta Kamiya, Tadaomi Furuta, Nobuo Kitada, Shojiro A. Maki

**Affiliations:** 1 Environmental Management Research Institute, National Institute of Advanced Industrial Science and Technology (AIST), Tsukuba 305-8569, Japan; 2 Department of Engineering Science, Graduate School of Informatics and Engineering, The University of Electro-Communications, Chofu 182-8585, Japan; kamiya0801@uec.ac.jp (G.K.); kitada@uec.ac.jp (N.K.); 3 School of Life Science and Technology, Tokyo Institute of Technology, Yokohama 226-8501, Japan; furuta@bio.titech.ac.jp

**Keywords:** serum albumin, coelenterazine, luminescence, redshift, indicators

## Abstract

Albumin assays in serum are important for the prognostic assessment of many life-threatening diseases, such as heart failure, liver disease, malnutrition, inflammatory bowel disease, infections, and kidney disease. In this study, synthetic coelenterazine (CTZ) indicators are developed to quantitatively illuminate human and bovine serum albumins (HSA and BSA) with high specificity. Their functional groups were chemically modified to specifically emit luminescence with HSA and BSA. The CTZ indicators were characterized by assaying the most abundant serum proteins and found that the CTZ indicators S6 and S6h were highly specific to HSA and BSA, respectively. Their colors were dramatically converted from blue, peaked at 480 nm, to yellowish green, peaked at 535 nm, according to the HSA–BSA mixing ratios, wherein the origins and mixing levels of the albumins can be easily determined by their colors and peak positions. The kinetic properties of HSA and BSA were investigated in detail, confirming that the serum albumins catalyze the CTZ indicators, which act as pseudo-luciferases. The catalytic reactions were efficiently inhibited by specific inhibitors, blocking the drug-binding sites I and II of HSA and BSA. Finally, the utility of the CTZ indicators was demonstrated through a quantitative imaging of the real fetal bovine serum (FBS). This study is the first example to show that the CTZ indicators specify HSA and BSA with different colors. This study contributes to the expansion of the toolbox of optical indicators, which efficiently assays serum proteins in physiological samples. Considering that these CTZ indicators immediately report quantitative optical signals with high specificity, they provide solutions to conventional technical hurdles on point-of-care assays of serum albumins.

## 1. Introduction

Albumin is the most abundant serum protein, occupying more than 50% of the proteins found in plasma. Albumin is also responsible for transporting vitamins, enzymes, and hormones throughout the human body. Hence, abnormal levels of albumin in the blood reflect life-threatening illnesses. Hypoalbuminemia can be a sign of an underlying medical condition such as heart failure, liver disease, malnutrition, inflammatory bowel disease, infections, and kidney disease [1,2].

Albumin assays are especially important in the field of forensic medicine; for example, many medicolegal investigations require estimation of postmortem interval. The postmortem interval can be reliably estimated with albumin assays, because, after death, albumin levels in blood linearly decrease in 2 to 72 h [3].

Conventionally, albumin levels have been determined using various methods including immunoassays, colorimetric assays, and fluorometric assays:

Typical immunoassay systems consist of antihuman serum albumin (HSA) antibody, the fluorophore- or luminophore-labeled secondary antibody, and the specific detector [4]. As an example of interesting colorimetric assays, HSA levels were previously determined with transitional metals [5]. This assay was possible, because the N-terminus of HSA is the primary binding site for the transitional metals Co(II), Cu(II), and Ni(II) [6]. Gold nanoparticles in a fluidic microchannel device were also used for measuring BSA. This method allowed rapid protein detection within 30 min, relying on the fast aggregation of AuNPs and rapid loading of analytes [7]. Recently, HSA can be even determined with paper-based ratiometric fluorescence analytical devices for point-of-care testing [8]. Moreover, Nishihara et al. developed coelenterazine (CTZ) analogues for the quantitative imaging of HSA. They showed that their CTZ analogues can illuminate HSA with a high specificity [9].

Conventionally, nCTZ and its analogues have been used as the common substrate of marine luciferases. These luciferin–luciferase reactions generating luminescence have been utilized in bioassays as emerging optical readouts to interrogate biological processes in vivo and in vitro.

Luminescence has clear, distinctive advantages over other imaging modalities, that is, luminescence does not require excitation light-causing phototoxicity. However, it needs a specific substrate for light development [2]. In addition, luminescence allows very low background intensities, long linear dynamic ranges, and high signal-to-background (S/B) ratios [4]. Therefore, luminescence intensity has been considered to be more quantitative than other imaging modalities, such as fluorescence.

Because luciferin acts as the luminophore in the luciferin–luciferase reactions, many researchers have chemically modified the native luciferins to improve the optical intensities and color variations [10,11]. The authors also developed many CTZ analogues through chemical synthesis and named K-, C-, and S-series CTZ analogues in our precedent studies [12,13,14].

In this present study, new luminescent indicators are created for the specific imaging of human and bovine serum albumins (HSA and BSA). The indicators were synthesized by modifying the functional groups of native coelenterazine (nCTZ) to specifically recognize HSA and BSA. The albumin specificity of the indicators was investigated using major serum proteins in the abundance ranking list. It was found that the indicators immediately luminesce blue and yellowish green with HSA and BSA, respectively. This shows that the origins of the albumins can be specified by their colors. The enzymatic kinetics of the albumin–indicator pairs were also measured to specify their kinetic constants. The albumin–indicator interactions were further confirmed with known specific inhibitors to the drug-binding sites (DBSs) of BSA. Finally, the utility of the CTZ indicators was demonstrated using quantitative imaging of the real fetal bovine serum (FBS).

This study shows that CTZ indicators are simple and label-free optical readouts, which can recognize specific albumins and quantitatively report the luminescence signals using specific colors. The CTZ indicators may be categorized into a “switch-on”-type tool to determine albumin levels in physiological samples. Furthermore, this study is an important addition to the conventional albumin assays, considering its importance in the diagnosis of related diseases in clinical and forensic medicine.

## 2. Materials and Methods

### 2.1. Reagents and Materials

The following serum proteins were obtained from Wako Pure Chemical Co. (Osaka, Japan): human serum albumin (HSA), bovine serum albumin (BSA), human immunoglobulin (HIgG), human γ-globulin (HγG), human transferrin (HTF), human fibrinogen (HFG), and soybean trypsin inhibitor (STI). In addition, the following serum proteins were purchased from Jackson Immuno Research (West Grove, PA, USA): bovine immunoglobulin (BIgG) and bovine γ-globulin (BγG). However, ovalbumin (OVA) and human fibronectin (HFN) were obtained from MP Biomedicals (Santa Ana, CA, USA) and CORNING (Corning, NY, USA), respectively.

The following candidate serum proteins were first selected based on their abundance in serum to illuminate them with our CTZ indicators: (i) human, bovine, and egg albumins (HAS, BSA, and OVA) were selected for the investigation, because they are the most abundant proteins in serum (35,000–52,000 mg/L) [15]; (ii) HIgG and BIgG were also selected, because they show the second-highest abundance among serum proteins (7000–16,000 mg/L); (iii) HTF was also chosen for the measurement, because it occupies the third abundant position among serum proteins (2000–3600 mg/mL); (iv) HγG and BγG were chosen, because γ-globulin grouping IgA, IgM, and IgY is the fourth abundant among serum proteins (i.e., 700–4000 mg/mL for IgA); (v) HFG was also selected, because it is the sixth abundant protein in serum; and (vi) STI and HFN were chosen for the measurement, because they are key components for the retardation of growth, digestive, and metabolic diseases [16] and human infective diseases [17], although they are the 19th and 26th abundant serum proteins, respectively.

Native coelenterazine (nCTZ) was purchased from NanoLights (Pinetop, AZ, USA). The CTZ indicators K6, S5, and S6 were obtained from our precedent studies [12,14]. The new CTZ indicators S5h and S6h were synthesized in this study.

### 2.2. Synthesis of New CTZ Indicators S5h and S6h for Selective Imaging of Serum Albumin

**Figure 1:**
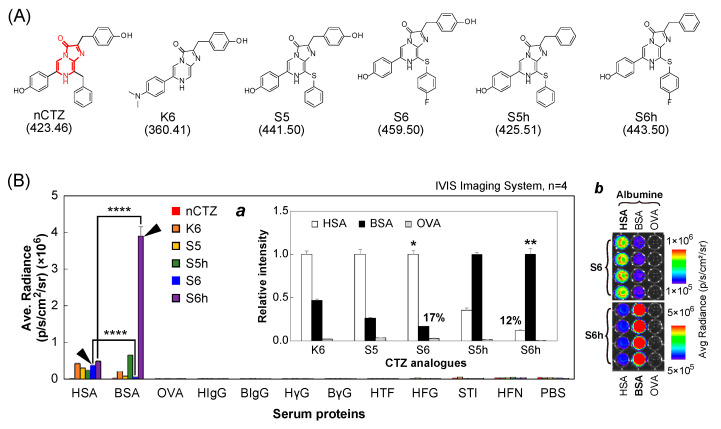
Coelenterazine indicators and their luminescence intensities in reaction with various serum proteins. (**A**) The chemical structures of native coelenterazine (nCTZ) and its indicators, which were used in this study. The numbers in parentheses indicate the molecular weights. The imidazopyrazinone backbone is highlighted in red. (**B**) Absolute luminescence intensities of the CTZ indicators in the reaction with various serum proteins. The arrowheads indicate the luminescence intensities showing the specificity of the CTZ indicators to respective serum proteins. The *p*-value (Student *t* test) is **** ≤ 0.0001. The inset ***a*** highlights the relative intensities of the CTZ indicators in response to serum albumins. The asterisks * and ** on the bars mark the HSA and BSA specificity of S6 and S6h, respectively. The inset ***b*** shows the corresponding optical images. Abbreviations: HSA, human serum albumin; BSA, bovine serum albumin; OVA, ovalbumin; HIgG, human immunoglobulin; BIgG, bovine immunoglobulin; HγG, human γ-globulin; BγG, bovine γ-globulin; HTF, human transferrin; HFG, human fibrinogen; STI, soybean trypsin inhibitor; HFN, human fibronectin; PBS, phosphate-buffered saline.

CTZ indicators S5h and S6h were synthesized based on the following scheme (Figure S1 and Supplementary Method S1). Compound **2** was synthesized through a Suzuki–Miyaura cross-coupling reaction of commercially available 2-amino-5-bromoaminopyrazine **1** with phenylboronic acid and tetrakis(triphenylphosphine)palladium (0). We further conducted a bromination using compound **2** with commercially available N-Bromosuccinimide to produce compound **3**. Compounds **4** and **5** were synthesized from compound **3** through benzenethiol or 4-fluorobenzenethiol substitution reaction with sodium hydride. Compounds **4** and **5** were then demethylated with boron tribromide to produce compounds **6** and **7**. Finally, compounds **6** and **7** with ketoacetal derivatives **12** were condensed and cyclized under hydrochloric acid conditions. The synthesized CTZ indicators were named S5h and S6h (Figure 1).

### 2.3. Specific Illumination of Serum-Abundant Proteins with Specific CTZ Indicators

The serum-abundant protein solutions were prepared through the dilution of their stocks with phosphate-buffered saline (PBS) to 1 mg/mL. Twenty µL of each solution were deployed into each well of the 96-well black-frame microplate (Thermo Fisher Scientific, Waltham, MA, USA). Each serum-abundant protein was deployed in four wells (*n* = 4).

Whereas, nCTZ and its indicators were initially dissolved with methanol and further diluted with PBS to 100 µM. Forty µL of the substrate solution were simultaneously injected into each well of the microplate using a multichannel micropipette (Gilson, Middleton, WI, USA). The corresponding luminescence intensities of the microplate were immediately determined using an IVIS Spectrum imaging system (PerkinElmer, Waltham, MA, USA) and analyzed using the Living Image version 4.7 software.

### 2.4. Luminescence Spectral Variation of CTZ Indicators Based on Serum Albumins

The luminescence spectra of the CTZ indicators were determined in the presence of serum albumins according to the following protocol.

The solutions of HSA and BSA were first prepared by dissolving with PBS to 10 mg/mL. Fifty µL of each solution were set in 200 µL PCR tubes, whereas the CTZ indicators were dissolved with methanol and further diluted with PBS to 100 µM. Fifty µL of the CTZ indicator solution was injected into each PCR tube. The corresponding luminescence spectra were immediately determined using a precision spectrophotometer (AB-1850, ATTO, Tokyo, Japan), which simultaneously measures all the wavelengths in the visible and NIR region. The integration time was five minutes.

Because S6h showed two different colors discriminating the origin of serum albumin, their spectral variance was further determined based on the mixing ratios of HSA and BSA according to the following protocol.

The HSA and BSA solutions were diluted with PBS to 5 and 0.2 mg/mL, respectively. These initial solutions were mixed in the following percentage ratios, that is, 100:0, 80:20, 60:40, 40:60, 20:80, and 0:100. Fifty µL of each albumin mixture were deployed in a 200 µL PCR tube, whereas the S6h substrate was initially dissolved with methanol and further diluted with PBS to 100 µM. Fifty µL of the substrate solution were injected into the PCR tube, and the corresponding luminescence spectra were determined using the precision spectrophotometer. The integration time was five minutes.

### 2.5. Determination of Kinetic Constants of CTZ Indicators Based on Serum Albumins

The representative kinetic constants of the albumin-specific CTZ indicators were determined in the presence of HSA or BSA according to the following protocol.

The following CTZ substrates were first dissolved with methanol and further diluted with PBS to 0, 2, 5, 10, 20, 30, 50, and 100 µM, that is, nCTZ, K6, S5, S5h, S6, and S6h. Forty µL of each substrate solution were deployed into each well of a 96-well black-frame microplate (Thermo Fisher Scientific). The microplate was then loaded in a microplate reader (TriStar2 S LB 942, Berthold, Germany), whereas HSA and BSA were diluted with PBS to 1.0 mg/mL. The albumin solutions were primed in the injectors of the microplate reader. After the programmed automatic injection of 40 µL of the albumin solutions into each well of the microplate, the corresponding luminescence intensities were determined with a 0.1 s integration and 20-point acquisition mode. The S6h–BSA kinetics study was repeated with the same protocol under the following concentration scale of S6h: 0, 10, 20, 50, 100, 200, 500, and 1000 µM.

The kinetic constants were calculated according to the following procedure: Because the BL intensities were reported in relative luminescence unit (RLU) over time, the RLU values were first converted to the corresponding photon counts per second before curve fittings, where the unique conversion ratio between RLU and photon count of the microplate reader was obtained using an LED standard light source (Hamamatsu Photonics, Shizuoka, Japan). The converted datasets were then plotted with a quadratic polynomial fitting tool of Excel (Microsoft, Seattle, WA, USA) to determine the initial velocities (*V*_0_). The obtained *V*_0_ values were deployed according to the concentrations of the CTZ indicators (substrate) in the datasheet of Prism version 9 software (GraphPad, Boston, MA, USA) and plotted for the determination of the corresponding kinetic parameters, *V*_max_, *K*_cat_, and *K*_m_.

### 2.6. Determination of the Wide-Range Dose–Response Curves of the Selected CTZ Indicators with Serum Albumins

The wide-range dose–response curves of the selected CTZ indicators were determined with varying concentrations of serum albumins according to the following protocol.

The HSA, BSA, and OVA solutions were first dissolved with PBS to 10 mg/mL. The stock solutions were consecutively diluted with PBS to the following concentrations: 0, 0.01, 0.02, 0.05, 0.1, 0.2, 0.5, and 1 mg/mL. Forty µL of each concentration of the dilutions were orderly deployed into each well of a 96-well black-frame microplate (Thermo Fisher Scientific), whereas the CTZ indicators K6, S6, and S6h, excluding nCTZ, were dissolved with methanol and further diluted with PBS to 20 µM. Forty µL of each CTZ indicator solution were simultaneously injected into the microplate using the 12-channel micropipette. The corresponding luminescence intensities of the microplate were immediately determined using the IVIS Spectrum imaging system.

### 2.7. Competition Studies between CTZ Indicators and Inhibitors at the Interaction Sites of HSA and BSA

A competition study was conducted to specify the specific interaction sites of HSA and BSA with the CTZ indicators according to the following protocol.

First, fatty-acid-free HSA and BSA solutions were prepared by diluting them with PBS to 1.0 mg/mL. Forty µL of each HSA and BSA solution were then premixed with 20 µL of 0, 1, 5, 10, 20, 40, 80, or 100 µM of warfarin for blocking DBS I of the albumins or of ibuprofen for blocking DBS II of the albumins before reacting with S6 or S6h. The premixtures were then orderly deployed into each well of a 96-well black-frame microplate (Thermo Fisher Scientific) and then simultaneously injected with 40 µL of 50 µM of S6 or S6h solutions using the 12-channel micropipette. The luminescence images were obtained using the IVIS Spectrum imaging system.

The inhibition kinetics was detailed using a Lineweaver–Burk analysis to highlight the competitive inhibition of ibuprofen with the substrate S6h according to the following protocol.

The S6h substrate was initially dissolved in methanol and diluted with PBS to 20, 40, 50, 80, and 100 µM. Forty µL of each S6h solution were premixed with 20 µL of 0, 10, or 50 µM ibuprofen solution. The mixtures were set in a 96-well black-frame microplate in order, whereas BSA was dissolved with PBS to 100 μg/mL. Forty µL of each BSA solution were then simultaneously injected into the wells of the microplate using a 12-channel micropipette. The luminescence images were obtained using the IVIS Spectrum imaging system.

### 2.8. Computational Molecular Docking of S6h and S6 into the BSA

The structure of BSA was obtained from the protein data bank (ID: 4OR0) [18], and the residues in the DBS II were relaxed after manually inserting the S6 into the DBS (replacing it with the bound naproxen). Then, the molecular docking was conducted to model the binding modes of S6 and S6h in BSA using the LibDock module of the BIOVIA Discovery Studio 2017 R2 (Dassault Systèmes; Vélizy-Villacoublay, France). Two and three poses were obtained for S6 and S6h, respectively. Among them, the top-ranked pose for each is described in the results.

This computational molecular docking was conducted only with BSA, because BSA binds S6h with the DBS II site alone according to the inhibition study in Section 2.7. Conversely, HSA appeared to be complex in binding with the CTZ indicators. For simplicity, we preferably chose the BSA–S6h pair in the docking simulation in this study.

### 2.9. Determination of Real Fetal Bovine Serum with S6h Indicators

The BSA specificity of S6h indicators was determined using the real fetal bovine serum (FBS) according to the following protocol.

The intact FBS was first diluted with PBS to the following percentages: 0, 0.005, 0.01, 0.05, 0.1, 0.5, 1, 5, and 10%. Forty µL of each solution per well were prepared in a 96-well black-frame microplate (Thermo Fisher Scientific), whereas the S6 and S6h solutions were diluted with PBS to 100 µM. Forty µL of each substrate solution per well were then simultaneously injected into the microplate using a 12-channel micropipette. The luminescence images were obtained using the IVIS Spectrum imaging system.

## 3. Results and Discussion

### 3.1. Design of CTZ Indicators for Albumin

In this study, the CTZ indicators, S5, S5h, S6, and S6h, were designed under the following consideration: To date, the functional group at the C-8 position of CTZ was less investigated. However, the authors speculated that the C-8 position of CTZ is the key for the specificity with luciferases and redshifts in the BL spectra. Specifically, the carbon ankle of the benzyl group at the C-8 position was replaced with sulfur (S) for extending the *π*-electron conjugation. In addition, the p-position of the benzyl structure at the C-8 position was optionally modified with fluorine (F).

In addition, Nishihara et al. previously reported that the hydroxy group (OH) at the C-2 position of CTZ is the key for the specific binding to Renilla luciferases or ALucs [19]. This is why we optionally introduced the OH group at the p-position of the benzyl group of the C-2 position.

### 3.2. Some CTZ Indicators Show Signature Luminescence Intensities with Specific Albumins

The optical reactivities of CTZ indicators with major serum proteins were investigated (Figure 1 and Figure S2).

The results in Figure 1B show that nCTZ was nonreactive with all the tested serum proteins. Conversely, all the CTZ indicators K6, S5, S5h, S6, and S6h emitted considerable intensities of luminescence with both HSA and BSA. Among them, S6h specifically reacted with BSA and generated 8.3-fold stronger luminescence intensities with BSA than with HSA (ca. 12%), whereas S6 selectively reacted with HSA and generated 5.9-fold stronger luminescence intensities than with BSA (ca. 17%). Similarly, S5h was more reactive with BSA than with HSA, whereas S5 luminesced more selectively with HSA than with BSA. However, all the tested CTZ indicators were not reactive with OVA and other serum proteins.

The specificity profiles strongly suggest that the hydroxyl group (OH) at the C-2 position of the CTZ indicators is a determinant of the specificity to serum albumins, considering that the only differences of S5 and S6 from S5h and S6h are the OH group at the C-2 position. The structural comparison between S5h and S6h also reveals that the fluoro (F) group at the C-8 position enhances the specificity, considering that the F group is the only difference between S5h and S6h. The absolute intensities of the S6h–BSA combination were minimally 8-fold superior to the other combinations.

### 3.3. The Luminescence Spectra Show That the CTZ Indicators Luminesce with BSA and HSA in Different Colors

**Figure 2:**
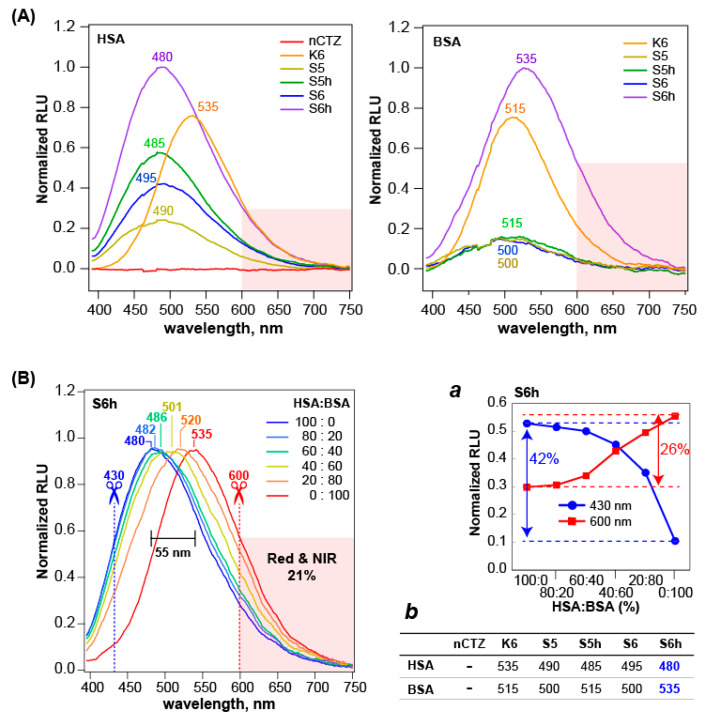
Luminescence spectra of CTZ indicators with HSA or BSA and the properties in their mixtures. (**A**) The luminescence spectra of the CTZ indicators with HSA (left) or BSA (right). The numbers denote the peak wavelengths of the spectra. (**B**) The normalized luminescence spectra of S6h in response to the HSA and BSA mixtures in different ratios. The numbers denote the peak wavelengths of the spectra. Inset ***a*** shows the varying luminescence intensities of the normalized spectra at 430 nm and 600 nm based on the mixing ratios. The percentages indicate the maximal intensity variances of S6h by changing the mixing ratios of HSA and BSA. Inset ***b*** summarizes the λ_max_ of the luminescence spectra of the CTZ indicators with HSA and BSA.

The luminescence spectra of the CTZ indicators were distinctive from each other in the λ_max_ values and peak heights (Figure 2).

The highest peak height was obtained with the S6h–HSA combination, followed by the S6h–BSA combination (Figure 2A). The colors of S6h were blue-peaked at 480 nm with HSA and yellowish-green-peaked at 535 nm with BSA, respectively. The gap between the peaks was ca. 55 nm. Conversely, S6 did not show considerable color changes when reacted with HSA and BSA. The colors with HSA and BSA were commonly green and peaked at around 490 and 510 nm, respectively. The second-largest gap, 30 nm, between the spectra of HSA and BSA was obtained with S5h. All the S-series CTZ indicators commonly emitted shorter and longer wavelength peaks with HSA and BSA, respectively. However, this feature conversed with K6, which emitted luminescence spectra peaked at a longer wavelength with HSA (535 nm) and shorter with BSA (515 nm).

Influenced by the dramatic color changes of S6h with HSA and BSA, we determined whether the spectral peaks of S6h were variable based on the mixing ratios of HSA and BSA solutions (Figure 2B). The results showed that the peak of S6h with 100% HSA at 480 nm was gradually shifted to the longer wavelengths (up to 535 nm) by increasing the ratios of BSA. The most redshifted peak was obtained with 100% BSA solution, in which the red portion longer than 600 nm in the spectrum occupied ca. 21% of the total area of the spectrum. When we cut the intensity heights at 430 and 600 nm, the heights at 430 nm were gradually decreased by improving the BSA portions in the mixture and finally lost 42% of the initial intensity, whereas those at 600 nm were increased up to 26% of the initial intensity by enhancing the BSA portions in the mixture (Figure 2B, inset ***a***).

The overall results in Figure 2B show that contamination of the serum samples can be easily judged with the colors, because the spectral peaks reflect the mixing ratios. This feature is especially important for forensic medicine to check whether or not the blood samples are contaminated.

The blue-to-redshifted peaks may be explained by the different energy levels of the intermediates of S6h upon reaction with BSA. The nCTZ generates four different energy levels of the intermediates, that is, neutral species, amide anion, phenolate anion, and pyrazine anion, showing broad emission peaks from blue to green (400–535 nm) [20]. In the case of S6h, it contains a longer π conjugation because of the sulfur elbow at the C-8 position compared with nCTZ. Such a role of the sulfur elbow was discussed in our previous study [14]. This feature should contribute to the redshifted luminescence of the intermediates. In addition, the blue- to redshifts of luminescence spectra of S6h based on the HSA–BSA ratios strongly suggest that the major intermediates generated by HSA and BSA are different. S6h generates two different, major intermediates because of the difference in the enzymatic properties inside the active sites of the HSA and BSA.

### 3.4. The Kinetic Study Explains Why S6 Is Specific to HSA, and S6h Is the Brightest with BSA

**Figure 3:**
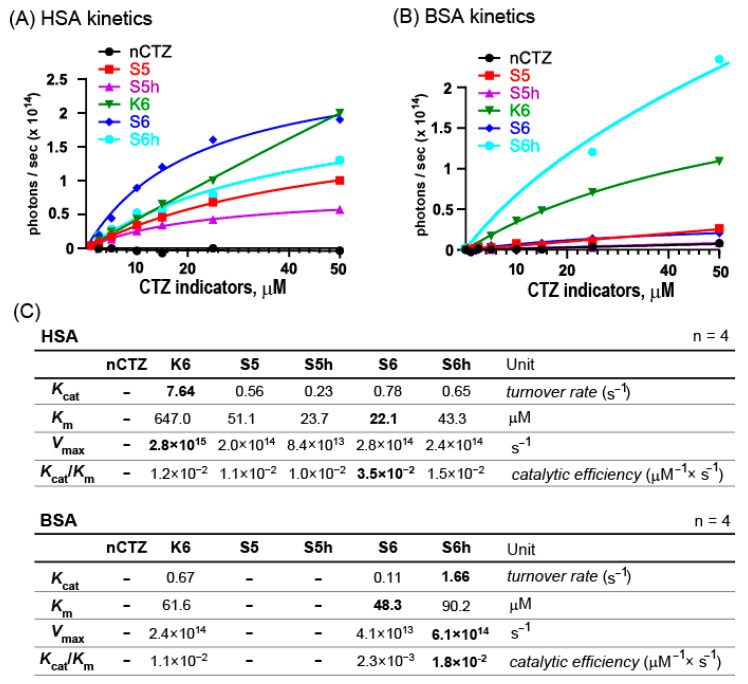
Determination of kinetic constants of CTZ indicators with HSA and BSA. (**A**) The initial luminescence intensities of the CTZ indicators in response to various concentrations of HSA. (**B**) The initial luminescence intensities of the CTZ indicators in response to various concentrations of BSA. (**C**) Summary of the kinetic constants showing the enzymatic properties of HSA and BSA in reaction with the CTZ indicators.

The enzymatic performances of the CTZ indicators were determined with respect to the kinetic constants (Figure 3).

The lowest Michaelis–Menten constant (*K*_m_), 22.1 μM, was obtained through the S6–HSA combination, followed by the S5h–HSA combination, that is, 23.7 μM. These values indicate that S6 and S5h substrates bind HSA with the strongest binding affinities compared with the others.

The turnover rate (*K*_cat_) and the maximal velocity (*V*_max_) were greatest with the K6–HSA combination, that is, 7.64 s^−1^ and 2.8 × 10^15^ s^−1^, respectively. However, the *K*_m_ value of the K6–HSA combination was the poorest among the tested. Collectively, the catalytic efficiency of the K6–HSA combination was found to be 1.2 × 10^−2^ μM^−1^s^−1^, which is less than that of the S6–HSA combination, that is, 3.5 × 10^−2^ μM^−1^s^−1^.

Comparing the S6–HSA combination with the S6–BSA combination, the catalytic efficiency of the S6–HSA pair (3.5 × 10^−2^ μM^−1^s^−1^) is more than 15-fold greater than that of the S6–BSA pair (2.3 × 10^−3^ μM^−1^s^−1^). This large difference in the catalytic efficiency is mostly caused by the difference in the *K*_cat_ values, that is, 0.78 versus 0.11 s^−1^. This comparison justifies that S6 is highly specific and bright with HSA rather than with BSA.

The S6h–BSA combination exhibited the highest brightness, as shown in Figure 1. This may be explained by the highest *K*_cat_ value of 1.66 × s^−1^ of the S6h–BSA combination, which is approximately 15-fold greater than that of the S6–BSA combination. The highest value in the turnover rate (*K*_cat_) means that BSA rapidly consumes S6h substrate, which is more than the other substrates. Interestingly, HSA consumes a less amount of S6h per second than BSA, that is, 0.65 s^−1^. This comparison explains that the S6h–BSA pair can be brighter than the other pairs if enough S6h is supplied.

The overall results confirm that HSA and BSA act as pseudoluciferases in the assay systems. These results are not surprising, considering the multifaceted enzyme roles of albumins in the human body, such as esterase, enolase, glucuronidase, peroxidase, aldolase, RNA-hydrolyzing agent, and antioxidant [21].

### 3.5. Wide-Range Dose–Response Curves Show the Albumin Specificity and the Luminescence Intensity of CTZ Indicators according to the Albumin Levels

**Figure 4:**
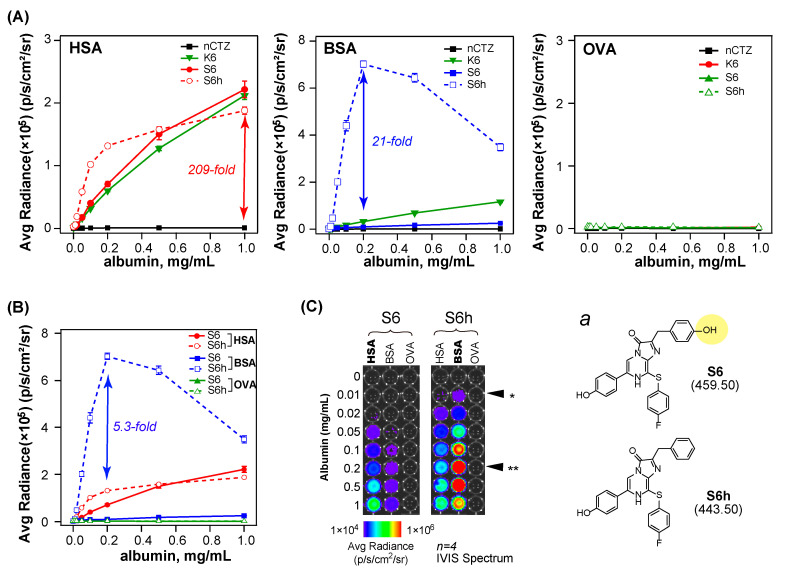
Wide-range dose–response properties of selected CTZ indicators at varying concentrations of serum albumin. (**A**) The wide-range dose–response curves of the selected CTZ indicators in response to the varying concentrations of HSA, BSA, and OVA. The arrows highlight the intensity gaps between the CTZ indicators, K6, S6, and S6h, which were reactive with HSA in the applied concentration range of 0–1 mg/mL. However, S6h shows relatively superior luminescence intensities with BSA compared to other CTZ indicators. (**B**) The summarized dose–response curves of S6 and S6h with varying concentrations of serum albumins. (**C**) The corresponding optical images of S6 and S6h in the reaction with varying concentrations of serum albumins. The arrowheads with asterisks * and ** indicate the detection limit and peak points, respectively. Inset ***a*** shows the chemical structures of S6 and S6h. The yellow shadow highlights the characteristic functional group of S6, which is different from that of S6h.

The wide-range dose–response curves of the selected CTZ indicators were investigated using varying concentrations of serum albumins (Figure 4).

Even by increasing the HSA concentrations, nCTZ did not show a significant elevation of the luminescence intensities (Figure 4A). Conversely, K6, S6, and S6h rapidly enhanced the luminescence intensities in an albumin-concentration-dependent manner. K6, S6, and S6h elevated the luminescence intensities by increasing the HSA levels, but S6h showed the sharpest enhancement in the lower concentration range of HSA, which is 0–0.2 mg/mL.

By increasing the BSA levels, S6h emitted the highest luminescence intensities, which were ca. 21-fold brighter than the K6–BSA combination and as the second-highest luminescence emitter at the 0.2 mg/mL BSA point. The luminescence intensities then drop in the BSA concentration range of 0.5–1.0 mg/mL. These luminescence intensity drops may be explained by the rapid turnover rates of BSA with S6h, as specified in Figure 3C. Considering the most rapid *K*_cat_ values of the S6h–BSA pair, the dropped luminescence intensities in the higher concentration range showed that BSA consumed all the S6h substrates applied and reached dim before the measurement.

Despite increasing the OVA levels, none of the CTZ indicators emitted considerable luminescence intensities in the tested concentration range, that is, 0–1.0 mg/mL.

The serum albumin specificities of S6 and S6h are summarized in Figure 4B. The S6h–BSA pair emitted ca. 5.3-fold stronger luminescence intensities than the S6h–HSA pair at the 0.2 mg/mL concentration point of the serum albumins. These albumin specificities of the CTZ indicators were visually observed in Figure 4C.

### 3.6. HSA and BSA Show Mixed Inhibition Chemistries with CTZ Indicators and Inhibitors

**Figure 5:**
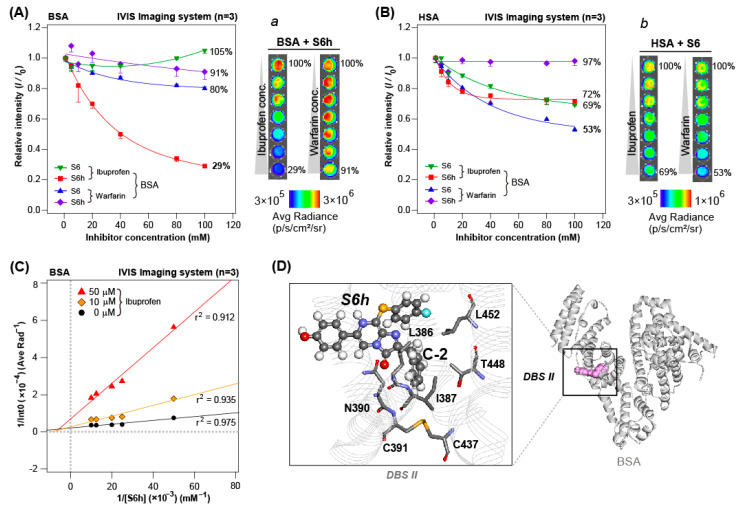
Competition of CTZ indicators against the inhibitors ibuprofen and warfarin and the drug-binding site (DBS) II in BSA. (**A**) Competition between the CTZ indicators and the inhibitors for the specific DBSs of BSA. The percentages show the remaining final intensity ratios of the CTZ indicators in the presence of 100 μM inhibitors, compared with those in the absence of the inhibitors ibuprofen and warfarin. Inset ***a*** shows the corresponding luminescence images. (**B**) Competition between the CTZ indicators and the inhibitors for the specific DBSs of HSA. The percentages show the remaining intensities after inhibition of 100 μM of the inhibitors, compared with those in the absence of the inhibitors. Inset ***b*** indicates the corresponding luminescence images. (**C**) Lineweaver–Burk inhibition plots show the inhibitory effects of ibuprofen on the reaction of S6h with BSA. The term r^2^ is defined as the coefficients of determination in the linear regression. (**D**) Structures of BSA and S6h on the DBS II of BSA (with focus on the C-2 of S6h). The right panel image shows the whole structure of BSA (gray ribbon) and the DBS II. The docked S6h molecule is represented by pink spheres. The left panel shows the close-up view of DBS II. The S6h molecule and the surrounding residues of the C-2 position of S6h are represented by the ball-and-stick method and sticks, respectively.

Serum albumins have multiple DBSs [22]. In this study, the DBSs for the CTZ indicators were investigated through competition studies (Figure 5 and Figure S3).

Because warfarin and ibuprofen are known to block DBSs I and II of serum albumins, respectively, the fixed amount of HSA or BSA was pretreated with varying amounts of inhibitors ranging from 0 to 100 μM. The mixtures were then simultaneously injected with the CTZ indicators, and the corresponding luminescence intensities were determined.

This competition assay in Figure 5A reveals that the luminescence intensities developed by the S6h–BSA pair are efficiently inhibited by ibuprofen up to 71%, whereas the luminescence intensities of the other pairs were not significantly inhibited by ibuprofen or warfarin. These results suggest that DBS II of BSA is the right site for interacting with S6h. Conversely, the S6h–HSA pair was partly inhibited by ibuprofen up to 28%, whereas the same pair was not influenced by warfarin (Figure 5B). This result suggests that DBS II of HSA partly interacts with S6h, but DBS I is not related to S6h binding. Meanwhile, the S6–HSA pair was commonly inhibited by both ibuprofen and warfarin up to 31 and 47%, respectively. These results show that both DBSs I and II of HSA interact with S6, although DBS I is more likely to bind with S6.

Influenced by the efficient inhibition effect of ibuprofen to S6h–BSA interaction, a Lineweaver–Burk analysis was further conducted to confirm the inhibitory effects of ibuprofen to the S6h–BSA interaction (Figure 5C). The results showed that higher ibuprofen concentrations increase the slopes of the plot of 1/[S6h]. Their correlation coefficients were found to be around 0.912–0.975.

The Lineweaver–Burk plot shows that the three fitting lines intersect at different negative values on the x-axis (meaning −1/*K*_m_). Then, the y-section points (meaning 1/*V*_max_ values) were elevated by increasing the ibuprofen amounts. This result confirms that the competition mode between S6h and ibuprofen to the sites of BSA was rather “mixed inhibition.” This implies that the DBSs of BSA can simultaneously interact with both S6h and ibuprofen. It is even possible that the first ligand-bound BSA may differentiate its reactivity from the second ligand. This result regarding an S6h–ibuprofen competition is different from a recent study by Nishihara et al., which showed that their CTZ indicator HuLumino1 is “competitive” with ibuprofen to DBS II of HSA [9].

All results and references can be concluded as follows: (i) The ligand binding chemistry of BSA can be more complex than expected, which should not be solely interpreted as “competitive” but also as “noncompetitive” or “mixed” modes based on the ligands; and (ii) the first ligand binding of BSA can affect the second ligand binding of BSA, which means that even the order of the pretreatment of BSA with ligands can result in different consequences.

### 3.7. Molecular Dockings of S6h and S6 to BSA Show Their Structural Differences

To clarify the difference in binding of S6h and S6 to BSA, the molecular docking of the substrates S6h and S6 into the DBS II of BSA was conducted (Figure 5D).

The binding energy of S6h was 89.6 kcal/mol (absolute energy), which was higher than that of S6 (79.7 kcal/mol). This large difference of 9.9 kcal/mol suggests that S6h binds more specifically to BSA than S6. Moreover, in the resulting structure, the C-2 group of S6h was surrounded by hydrophobic residues (L286, I387, and L452) and a hydrophobic region of polar residues (N390, C391, C437, and T448; Figure 5D), showing that the docking pose of S6 was almost the same with that of S6h. This hydrophobic environment surrounding the C-2 position could influence the binding affinities of S6h and S6. In addition, we found that polar and charged residues (N390, R409, K413, R484, and S488) were located near the imidazopyrazinone backbone of CTZ indicators, which have potential to contribute to the generation of luminescence.

Regarding the difference between BSA and HSA, most of the residues around DBS II are well conserved. Of the residues surrounding the C-2 position above, only T448 in BSA was replaced by A449 in HSA. The bulkiness of this residue may affect binding, although distinct from its polarity effect. On the C-6 side, however, R412 in BSA was replaced by K413 in HSA. This slight difference in basic residues may influence the emitting species as described above. Furthermore, the K413 residue of BSA, which is conserved as K414 in HSA, interacted with the S atom (elbow) at the C-8 position, suggesting specificities with S-series substrates. This basic residue is likely responsible for the substrate binding and luminescence of the more luminescent S-series than the nCTZ.

### 3.8. Albumins in Real Fetal Bovine Serum Can Be Determined with the CTZ Indicators

**Figure 6:**
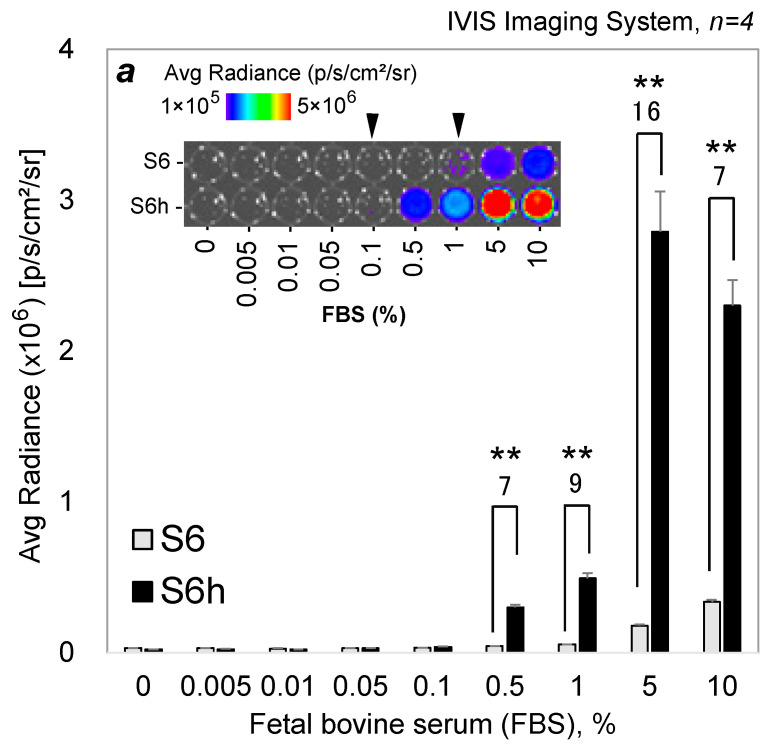
Dose-responsive luminescence intensities of S6 and S6h in response to real FBS. The *p*-value (Student *t* test) is ** ≤0.01. The numbers on the bars indicate the fold intensities of the luminescence of S6h over S6. Inset ***a*** shows the corresponding optical image. The arrowheads highlight approximate detection limits of the CTZ indicators.

Finally, the utility of the CTZ indicators was demonstrated using quantitative imaging of real FBS (Figure 6).

The results showed that S6h significantly elevated the luminescence intensities from the 0.1% point of FBS, but S6 started the considerable elevation at around the 1% point of FBS. S6h then rapidly elevated the luminescence intensities in the 0.5–10% FBS range, in which maximal luminescence intensity was observed at the 5% point of FBS. The luminescence intensity of S6h was ca. 16-fold greater than that of S6 at the 5% point of FBS. The greater BL intensities of S6h than those of S6 are interpreted as follows: the BSA as the major serum protein ingredients in FBS selectively reacted with S6h and generated stronger BL intensities than with S6. These biased BL intensities of S6h to BSA are supported by the kinetic study in Figure 3, where the BSA–S6h combination has better turnover rates and catalytic efficiency. Collectively, this large S/B ratio proves that S6h is a practical and efficient optical indicator for identifying BSA.

The dataset was also analyzed with respect to the statistical significance. The correlation coefficients (r^2^) between the applied concentrations of FBS and the BL intensities were found to be 0.999 and 0.884 with S6 and S6h, respectively. The significant differences between the two data groups by S6 and S6h were also determined with the unpaired Student *t* test (2-tailed) at the FBS levels of 0.5–10%. The results showed that the two groups by S6 and S6h are significantly different from each other, because the probability scores were 0.00011, 0.00011, 0.00028, and 0.00015 at the FBS levels of 0.5, 1, 5, and 10%, respectively.

These results convinced us to determine the origins and the albumin levels in blood with respect to forensic medicine, considering that the major protein of serums is albumin. The CTZ indicators can be applied for the estimation of postmortem interval, which is a very important question in many medicolegal investigations. The estimation of postmortem interval is possible, because, after death, albumin levels in blood linearly decrease in 2 to 72 h [3].

The present assay based on CTZ indicators has clearly distinctive advantages over other albumin assay modalities in the measurement time and simplicity: i.e., the present assay results can be obtained by a simple protocol, that is, just by mixing an aliquot of the indicator with albumins. Conversely, conventional immunoassays require a pretreatment of the samples and relatively long assay procedures [4]. Even advanced albumin assays using gold nanoparticles and fluidic microchannel devices takes 30 min [7]. Furthermore, the BL signals in our assay are self-generated by simply mixing the indicator with albumins, whereas fluorescence-based assays require external light sources and can be influenced by light attenuations and cross talks between the excitation and emission signals [8].

## 4. Conclusions

Considerable scientific evidence supports the importance of albumin measurement in serum for the prognostic assessment of many life-threatening diseases [1,2]. This present study demonstrated synthetic CTZ indicators that quantitatively report luminescence signals in response to human serum albumin (HSA) and bovine serum albumin (BSA), with high specificity and colorimetric variety. Their functional groups were chemically modified to specifically emit luminescence with HSA or BSA. For example, the presence or absence of the hydroxy group at the C-2 position of the indicators was a determinant of the selectivity to BSA and HSA, respectively. We proved that the CTZ indicators immediately emit “switch-on”-type luminescence signals with HSA and BSA. The colors changed significantly from blue to yellowish green depending on the type of serum albumin. The peak heights were quantitative and proportional to the HSA–BSA ratios. The kinetics studies and inhibition studies on HSA and BSA confirmed that serum albumins exert pseudoluciferase roles, catalyzing CTZ indicators as the substrates. Finally, the utility of the CTZ indicators was demonstrated using quantitative imaging of the real FBS.

The future studies of the present albumin assay system should be directed to the following ways: (i) the absolute intensities of S6 and S6h in the present study are unbalanced, as shown in Figure 1B. If the biased intensities are relieved, this assay system may be applicable to multireporter systems, and (ii) the colors of the CTZ indicators with albumins are currently limited in blue to yellowish green. More redshifted colors should diversify the color palette and improve the tissue permeation in animal models.

This study contributes to the expansion of the toolbox of optical indicators, which efficiently assay serum proteins in physiological samples with high specificity and colors. Considering that the CTZ indicators immediately report quantitative optical signals with high specificity, the indicators provide a solution to the conventional technical hurdles on point-of-care assays of serum albumins.

## Data Availability

The data presented in this study are available on request from the corresponding author.

## Supplementary Materials

The following supporting information can be downloaded at: https://www.mdpi.com/article/10.3390/s23136020/s1, Figure S1: Synthetic scheme for CTZ indicators; Figure S2: Whole-scale luminescence images of S6 and S6h in the reaction with various serum proteins; Figure S3: Full-scale luminescence images of the blocking effects of ibuprofen and warfarin to the DBSs of BSA and HAS; Supplementary Method S1. Specific procedures of organic syntheses and the corresponding NMR and Mass data for the CTZ indicators.
