# Supplementary material for: Coelenterazine Indicators for the Specific Imaging of Human and Bovine Serum Albumins
Source: Sensors (Basel). 2023 Jun 29;23(13):6020. doi: 10.3390/s23136020 (PMC10346935; doi:10.3390/s23136020)
Supplement: Supplementary file 1 [file sensors-23-06020-s001.zip › sensors-2436798-supplementary.pdf]

Supplementary Information

Coelenterazine Indicators for the Specific Imaging of Human and Bovine Serum Albumins

Sung-Bae Kim [1,*], Genta Kamiya [2], Tadaomi Furuta [3], Nobuo Kitada [2] and Shojiro A. Maki [2,*]

[1] Environmental Management Research Institute, National Institute of Advanced Industrial Science and Technology (AIST), Tsukuba 305-8569, Japan
[2] Department of Engineering Science, Graduate School of Informatics and Engineering, The University of Electro-Communications, Chofu 182-8585, Japan; kamiya0801@uec.ac.jp (G.K.); kitada@uec.ac.jp (N.K.)
[3] School of Life Science and Technology, Tokyo Institute of Technology, Yokohama 226-8501, Japan; furuta@bio.titech.ac.jp
* Correspondences: kimu-sb@aist.go.jp (S.-B.K.); s-maki@uec.ac.jp (S.A.M.)

Table of contents

Figure S1. Synthetic scheme for CTZ indicators……………………….……….…… page 2

Figure S2. Whole scale luminescence images of S6 and S6h in the reaction with various serum proteins……………………………………………………….……………..…page 3

Figure S3. Full-scale luminescence images of the blocking effects of ibuprofen and warfarin to the interaction sites of BSA and HSA……………..……….……………….page 3

Supplementary Method S1. Specific procedures of organic syntheses and the corresponding NMR and Mass data for the CTZ indicators……………………………..……..……...page 4

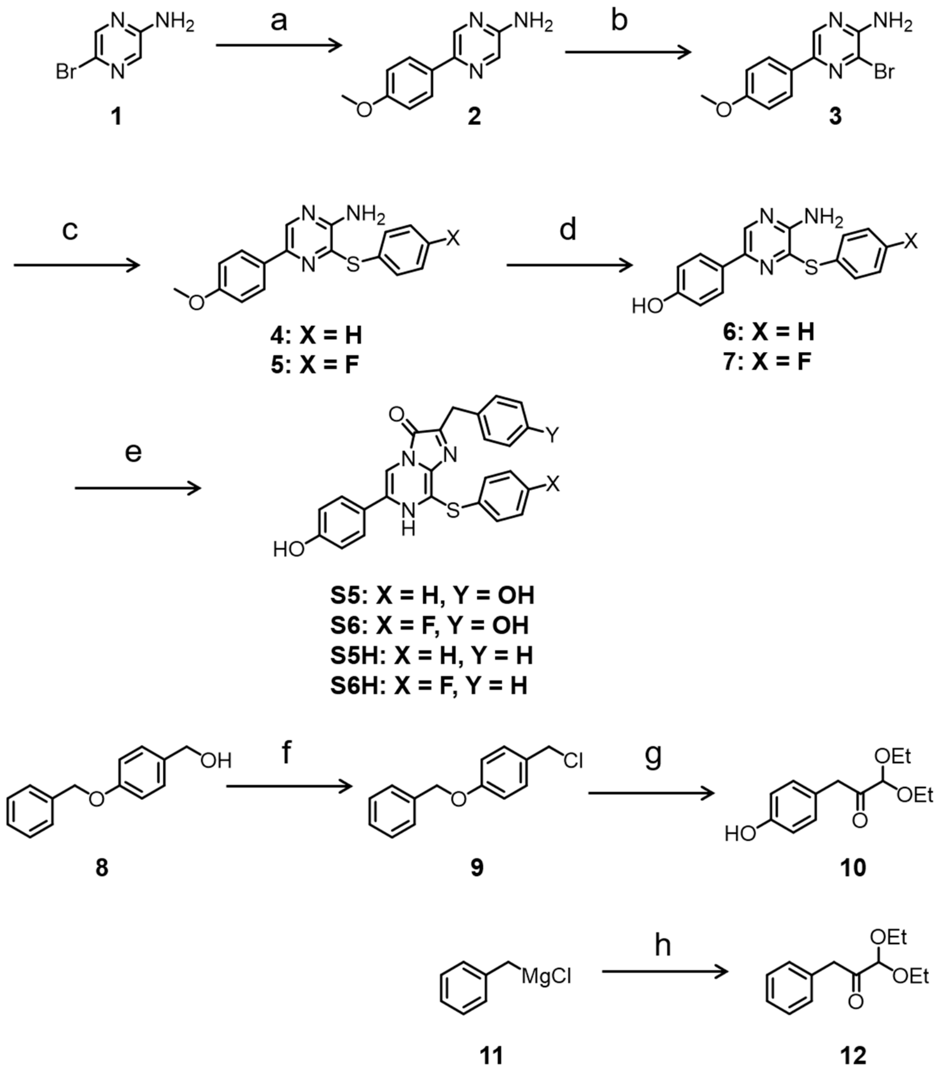

Figure S1. Synthetic scheme for nCTZ analogues.

a: Tetrakis(triphenylphosphine)palladium(0), 1,4-dioxane, 2 M Na_2CO_3 aq, 110 °C. b: *N*-Bromosuccinimide, chloroform, r.t. c: Sodium hydride, *N,N*-dimethylformamide, 0 °C to 110 °C. d: Boron tribromide, chloroform, -80 °C to r.t. e: 12 M HCl aq, eta, 60 °C. f: Thionyl chloride, dichloromethane, 0 °C. g: 1) Mg, 1,2-dibromoethane, ethyl diethoxyacetate, tetrahydrofuran, r.t. to reflux to 0 °C to -80 °C. 2) palladium-activated carbon, methanol, hydrogen, r.t. h: ethyl diethoxyacetate, tetrahydrofuran, -80 °C.

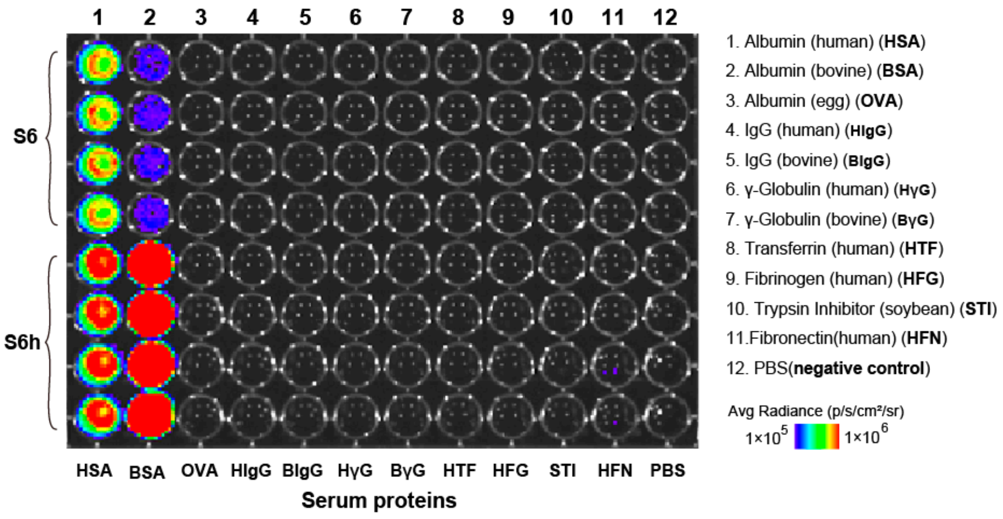

Figure S2. Whole scale luminescence images of S6 and S6h in the reaction with various serum proteins. Abbreviations: HSA, human serum albumin; BSA, bovine serum albumin; OVA, egg ovalbumin; HIgG, human immunoglobulin; BIgG, bovine immunoglobulin; HγG, human γ-globulin; BγG, bovine γ-globulin; HTF, human transferrin; HFG, human fibrinogen; STI, soybean trypsin inhibitor; HFN, human fibronectin; PBS, phosphate buffered saline.

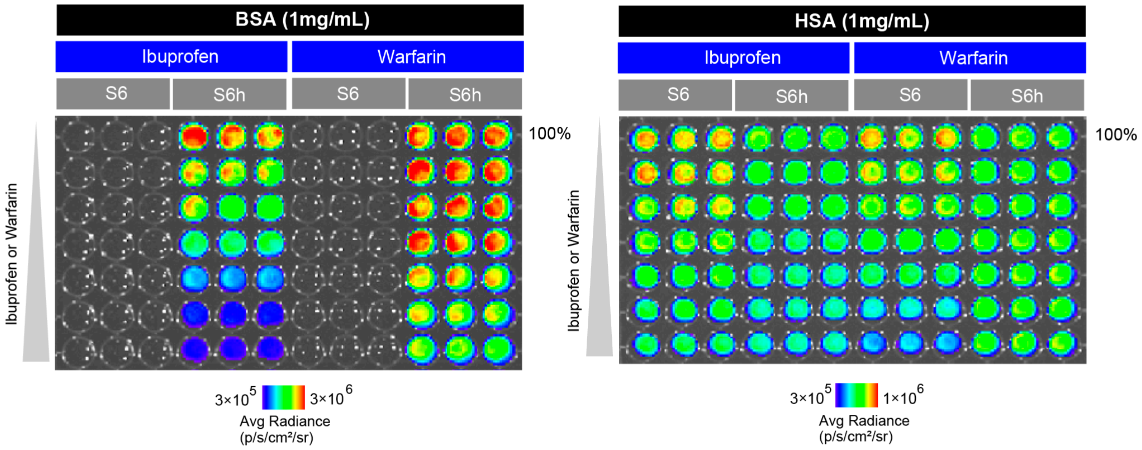

Figure S3. Full-scale luminescence images of the blocking effects of ibuprofen and warfarin to the interaction sites of BSA and HSA.

Supplementary Method S1. Specific procedures of organic syntheses and the corresponding NMR and Mass data for the nCTZ analogues.

Compounds **2-10**, **S5,** and **S6** were synthesized based on our previously reported procedures (Kamiya et al. *Int. J. Mol. Sci.* **2023**, *24*(2), 1420).

1,1-diethoxy-3-phenylpropan-2-one 12

Ethyl diethoxyacetate (1.24 mL, 7.0 mmol) was dissolved in dry THF (60 mL) and stirred at -80 °C under argon for 1 hour. Benzylmagnesium chloride (16 % in THF, 1 mol/L) (8.4 mL, 8.4 mmol) was fallen in drops in the cooled flask over 10 min. The mixture was stirred at –80 °C for 1 hour. After the reaction was completed, water was added, and the product was extracted with ethyl acetate (3 × 150 mL). The organic phase was dried over anhydrous sodium sulfate and evaporated. The obtained white oil substance was separated by column chromatography (hexane / ethyl acetate = 15/1 to 10/1) to obtain compound **12** (1.26 mg, 5.66 mol, 81%) as a colorless oil

^{1}H-NMR (500 MHz, Chloroform-*d*) δ 7.33-7.21 (m, 5H), 4.63 (s, 1H), 3.89 (s, 2H), 3.73-3.67 (m, 2H), 3.58-3.52 (m, 2H), 1.25 (t, J = 6.9 Hz, 6H)
^{13}C-NMR (126 MHz, Chloroform-*d*) δ 203.2, 133.8, 129.8, 128.5, 126.9, 102.4, 63.4, 43.7, 15.2
HR-MALDI-MS: *m/z*: $[M+H]^+$ calcd for $C_{13}H_{18}O_3Na$, 245.11482; found, 245.11490.

2-benzyl-8-((4-fluorophenyl)thio)-6-(4-hydroxyphenyl)imidazo[1,2-a]pyrazin-3(7H)-one S5h

Compound **6** (30 mg, 0.1 mmol) and compound **12** (34 mg, 0.15 mmol) were dissolved in ethanol at room temperature. 12M HCl aq (100 μL) was added to the mixture for 12 hours at 60°C under argon. The mixture was evaporated. The residue was separated by automated flash chromatography (Smart Flash EPCLC AI-580S, Universal Columns, chloroform /methanol = 99/1 to 90/15) to obtain analogue **S5h** (12.1 mg, 0.028 mmol, 28 %, reddish—brown solid).
^{1}H-NMR (500 MHz, Methanol-*d*$_4$) δ 8.43 (s, 1H), 7.70 (d, J = 6.9 Hz, 2H), 7.59-7.54 (m, 5H), 7.38-7.24 (m, 5H), 6.74 (dd, J = 6.6, 2.0 Hz, 2H), 4.26 (s, 2H)
HR-MALDI-MS: *m/z*: $[M+H]^+$ calcd for $C_{25}H_{20}N_3O_2S$, 426.12707; found, 426.12570.

Compounds **S6h** were prepared with a similar procedure to the preparation of **S5h**.

2-benzyl-8-((4-fluorophenyl)thio)-6-(4-hydroxyphenyl)imidazo[1,2-a]pyrazin-3(7H)-one S6h

15.5 mg, 0.034 mmol, 36 %, reddish—brown solid
^{1}H-NMR (500 MHz, Methanol-*d*$_4$) δ 8.48 (s, 1H), 7.76-7.73 (m, 2H), 7.60 (dd, J = 6.9, 1.7 Hz, 2H), 7.37-7.26 (m, 7H), 6.77 (dd, J = 6.9, 1.7 Hz, 2H), 4.29 (s, 2H)

HR-MALDI-MS: *m/z*: $[M+H]^+$ calcd for $C_{25}H_{19}N_3O_2FS$, 444.11765; found, 444.11706.

Reference

1. Kamiya, G.; Kitada, N.; Furuta, T.; Hirano, T.; Maki, S. A.; Kim, S. B., S-Series Coelenterazine-Driven Combinatorial Bioluminescence Imaging Systems for Mammalian Cells. *Int J Mol Sci* **2023,** 24(2), 1420.
